# Supplementary figures and images for: Separating random and deterministic sources of computational noise in explore-exploit decisions
Source: PLoS Comput Biol. 2026 Mar 17;22(3):e1014026. doi: 10.1371/journal.pcbi.1014026 (PMC13020999; doi:10.1371/journal.pcbi.1014026)

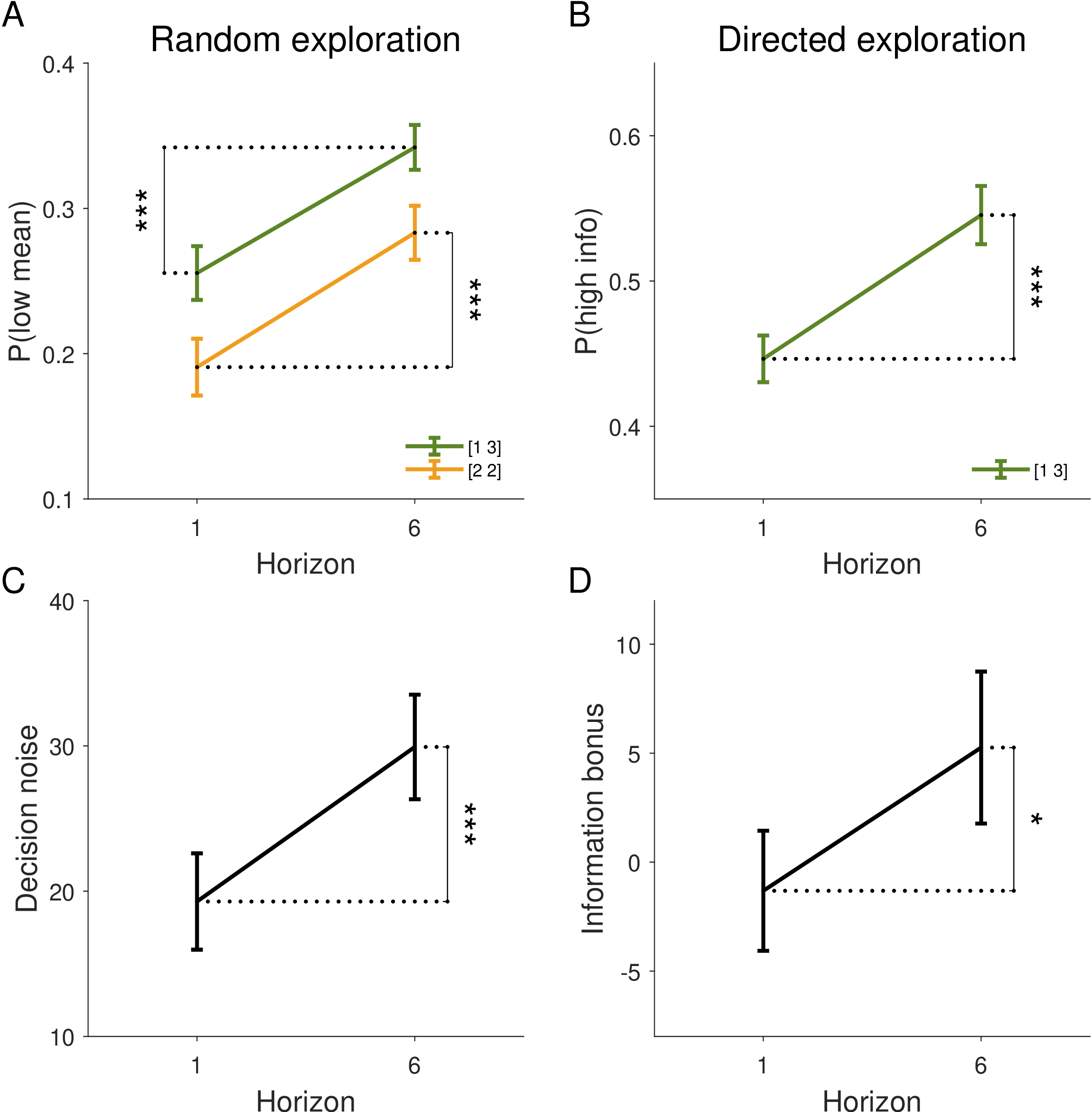

Supplement: S1 Fig — (A) model-free measure of behavioral variability, p(low mean, increases with horizon. (B) model-free measure of information seeking, p(high info, increases with horizon. (C) model-based measure of behavioral variability, decision noise σ, increases with horizon. (D) model-based measure of information seeking, information bonus A, increases with horizon. (TIFF) [file pcbi.1014026.s001.tiff]

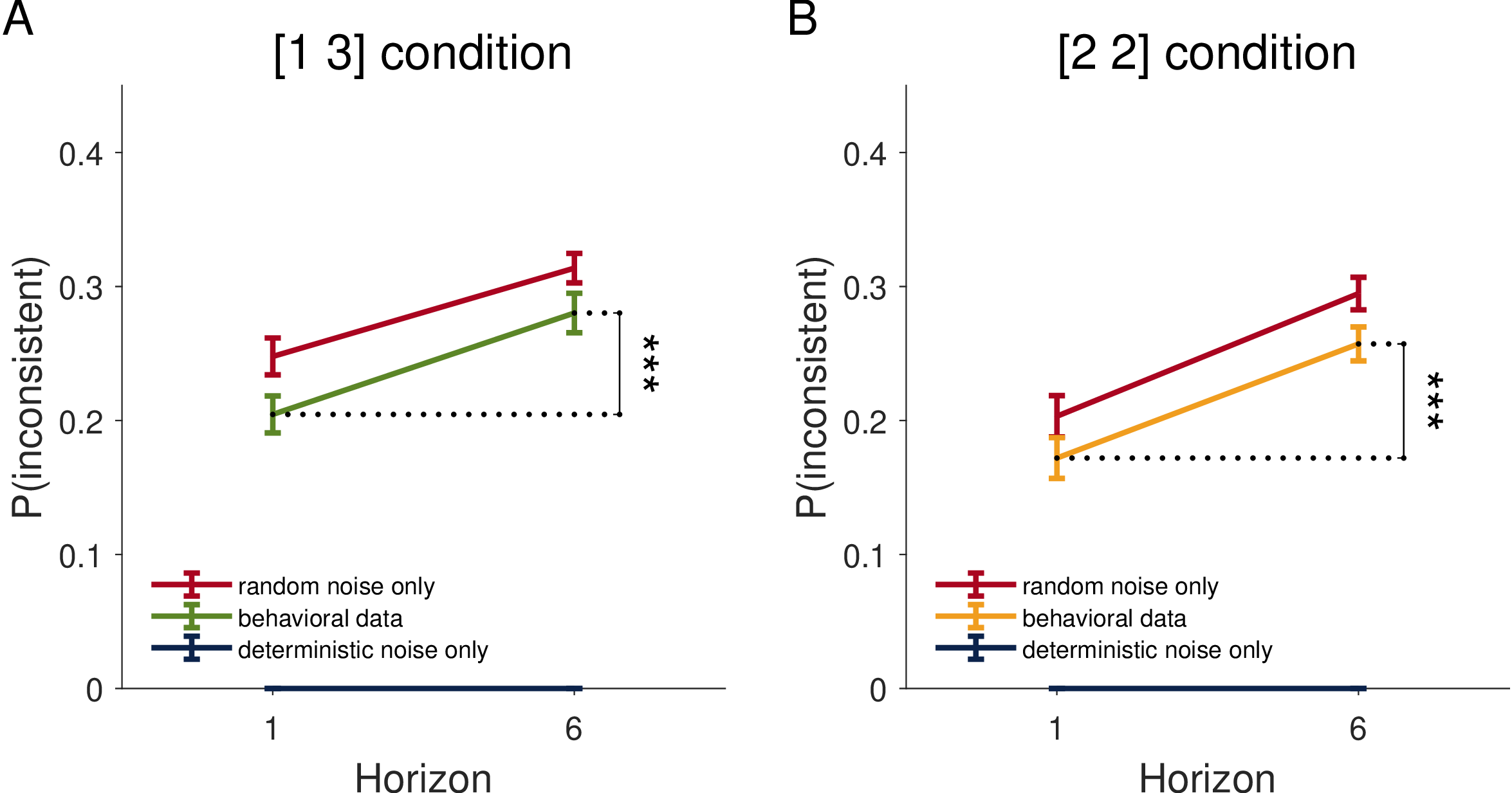

Supplement: S2 Fig — For both the [1 3] (A) and [2 2] (B) condition, people show greater choice inconsistency in horizon 6 than horizon 1. However, the extent to which their choices are inconsistent lies between what is predicted by purely deterministic and random noise, suggesting that both noise sources influence the decision. (TIFF) [file pcbi.1014026.s002.tiff]

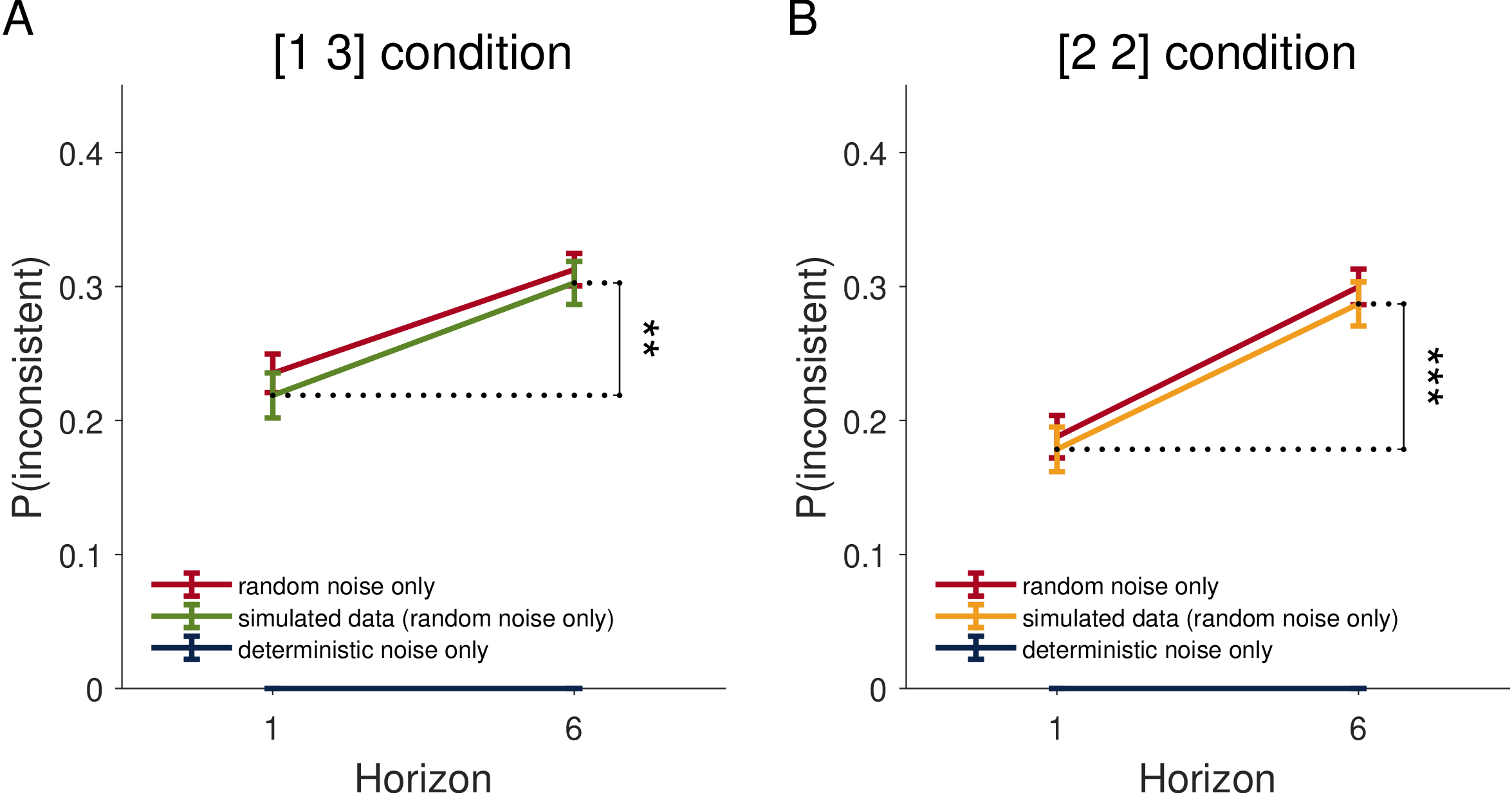

Supplement: S3 Fig — The extent to which simulated choices are inconsistent completely overlaps with our pure random noise prediction(p > 0.05). This suggests that when choice inconsistency lies below the pure random noise prediction indeed provides evidence that deterministic noise exists in random exploration (Fig 3). (TIFF) [file pcbi.1014026.s003.tiff]

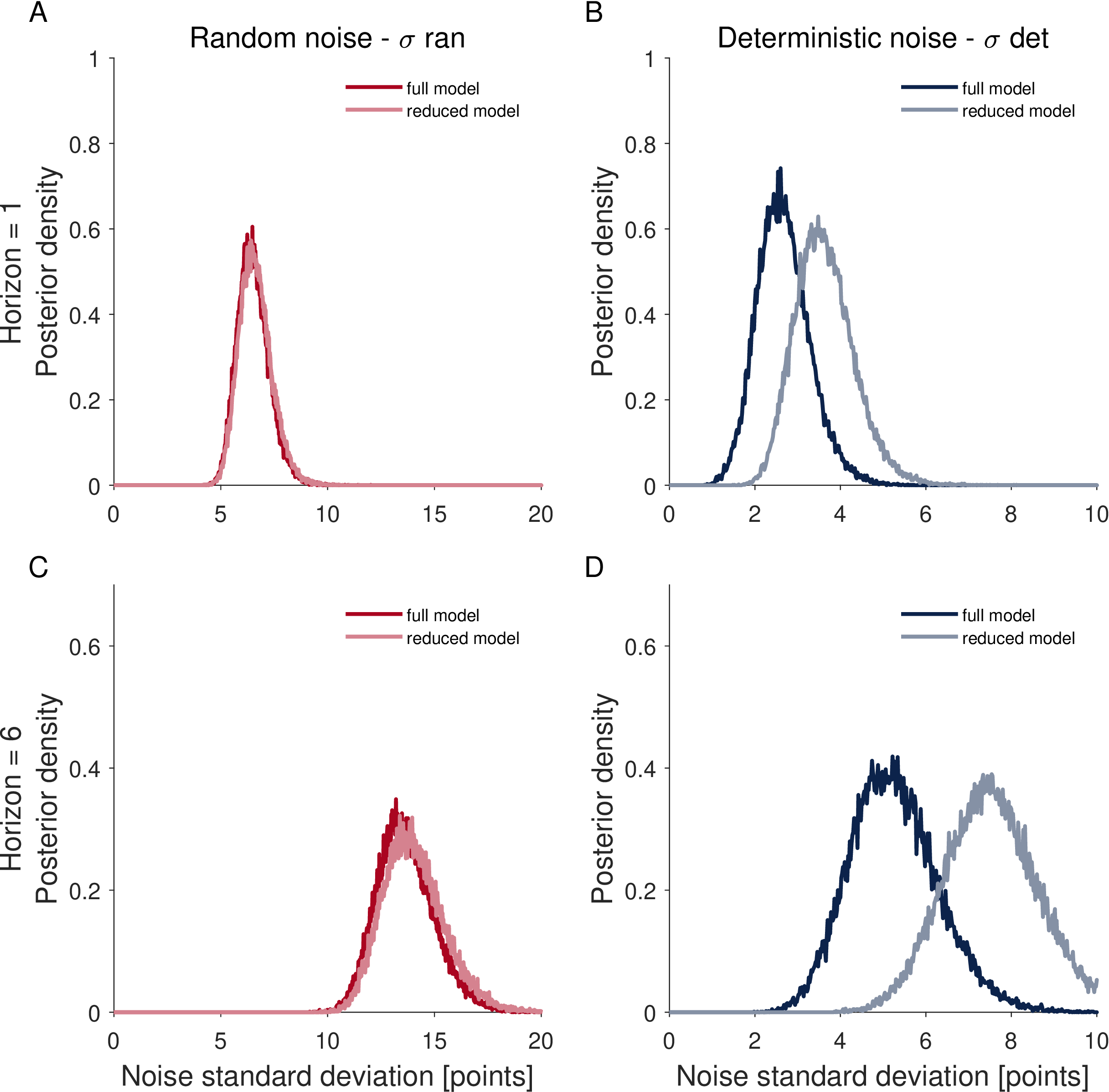

Supplement: S4 Fig — In the reduced model where the deterministic effect of uncertainty condition is omitted from the model, deterministic noise is higher compared to the full model that accounts for the effect of uncertainty. Random noise remains unchanged between the two models. (TIFF) [file pcbi.1014026.s004.tiff]

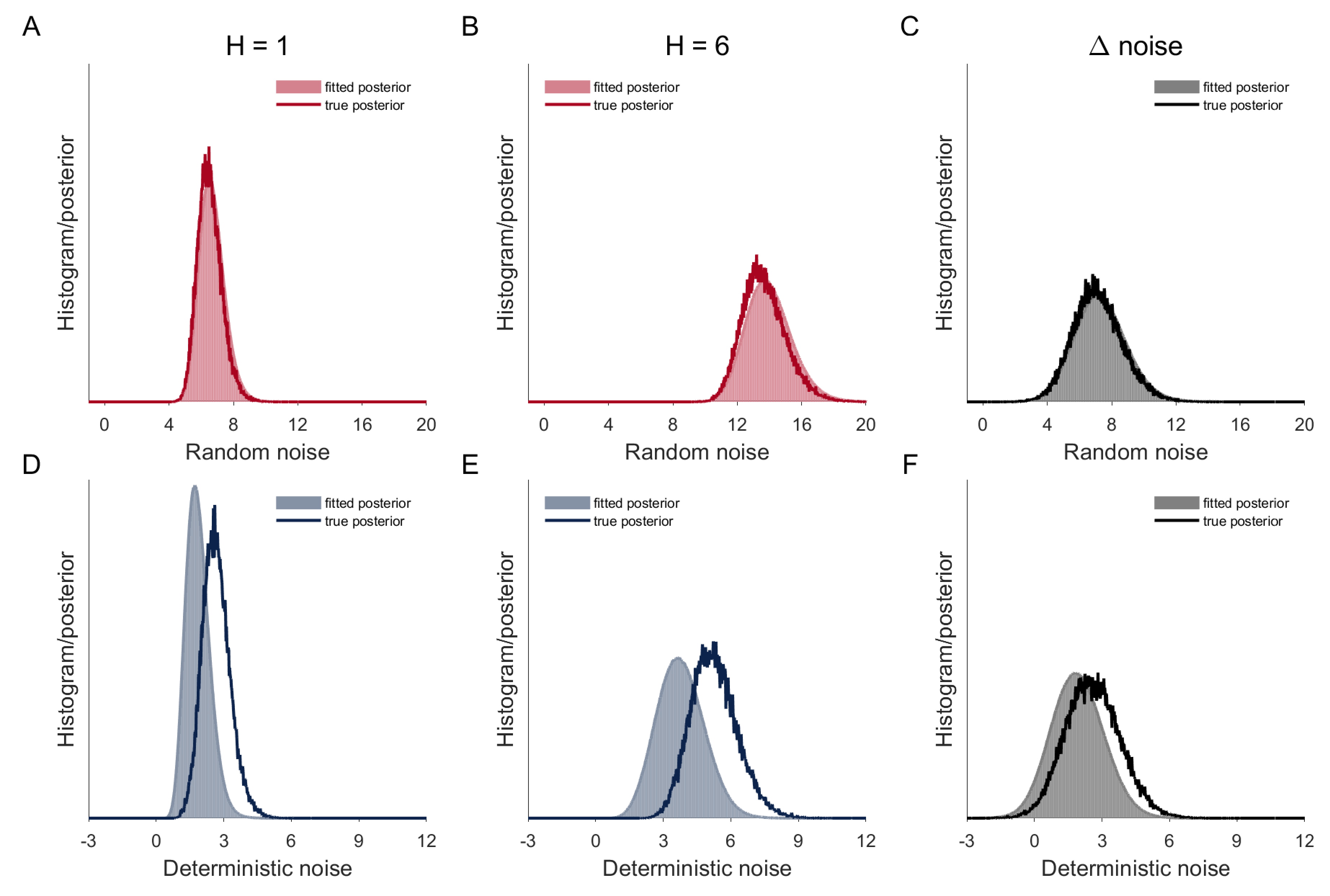

Supplement: S5 Fig — Parameter recovery over the posterior distribution of random and deterministic noise standard deviations σdet and σran. Solid lines are true posterior used to simulate choices. Lighter color shades represent the re-fitted posterior to the simulated choices. Our model fitting procedure faithfully recovers the non-stimulus-driven random noise (A, B), but systematically underestimates deterministic noise in both horizons (D, E). The horizon differences in random noise is also faithfully recovered (C). The horizon differences in deterministic noise is also underestimated but not significant (F). (TIFF) [file pcbi.1014026.s005.tiff]

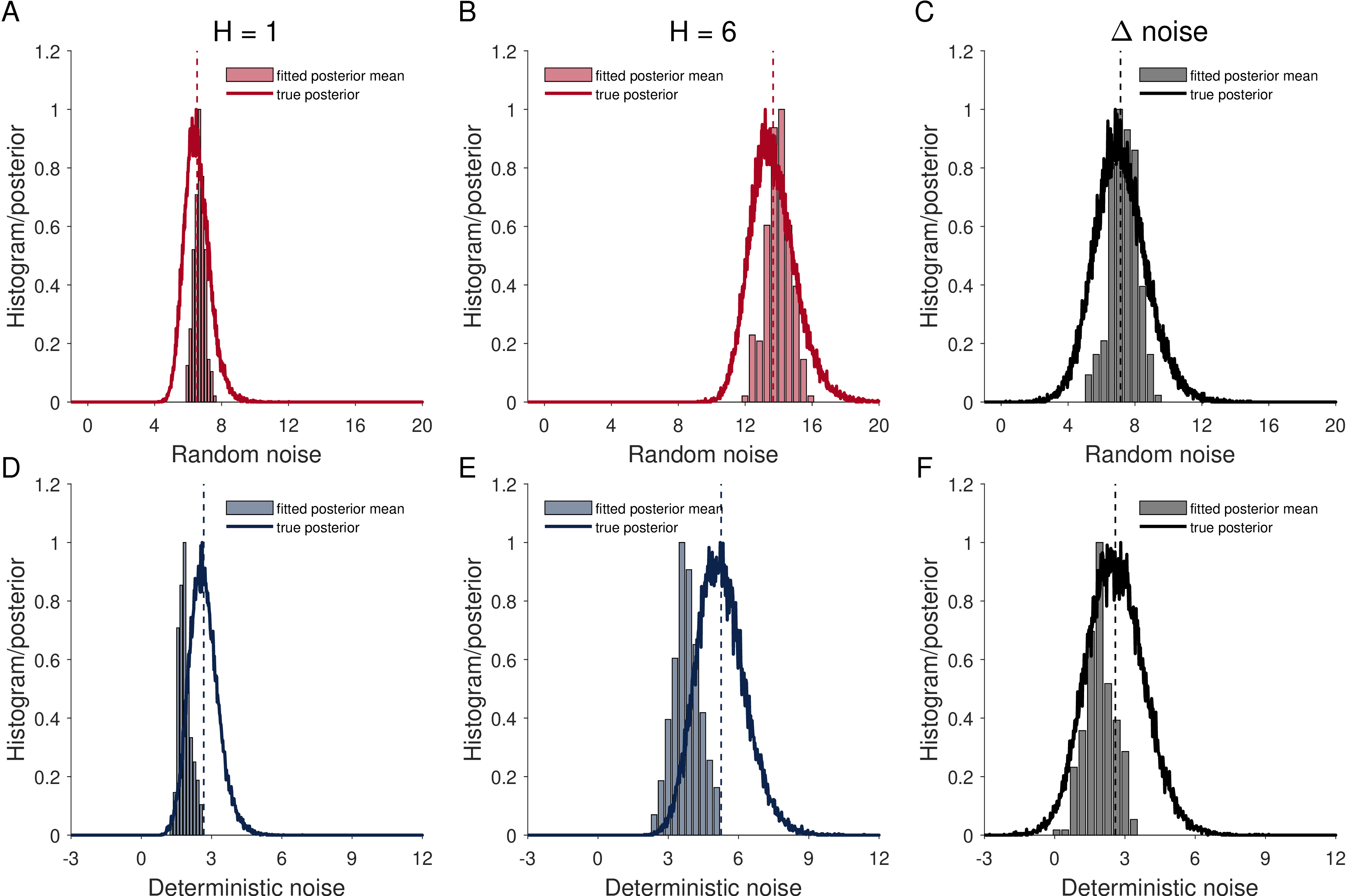

Supplement: S6 Fig — Parameter recovery over the mean estimates of random and deterministic noise standard deviations σdet and σran. Solid lines are true posterior used to simulate choices, dashed black line is the mean of the true posterior. Histograms represent the mean estimates of the respective parameters in the refitting to the simulated data. (A) and (B) are random noise at H = 1 and H = 6, respectively. (C) is the random noise differences between horizons. (D) and (E) are deterministic noise at H = 1 and H = 6, respectively. (F) is the deterministic noise differences between horizons. (TIFF) [file pcbi.1014026.s006.tiff]

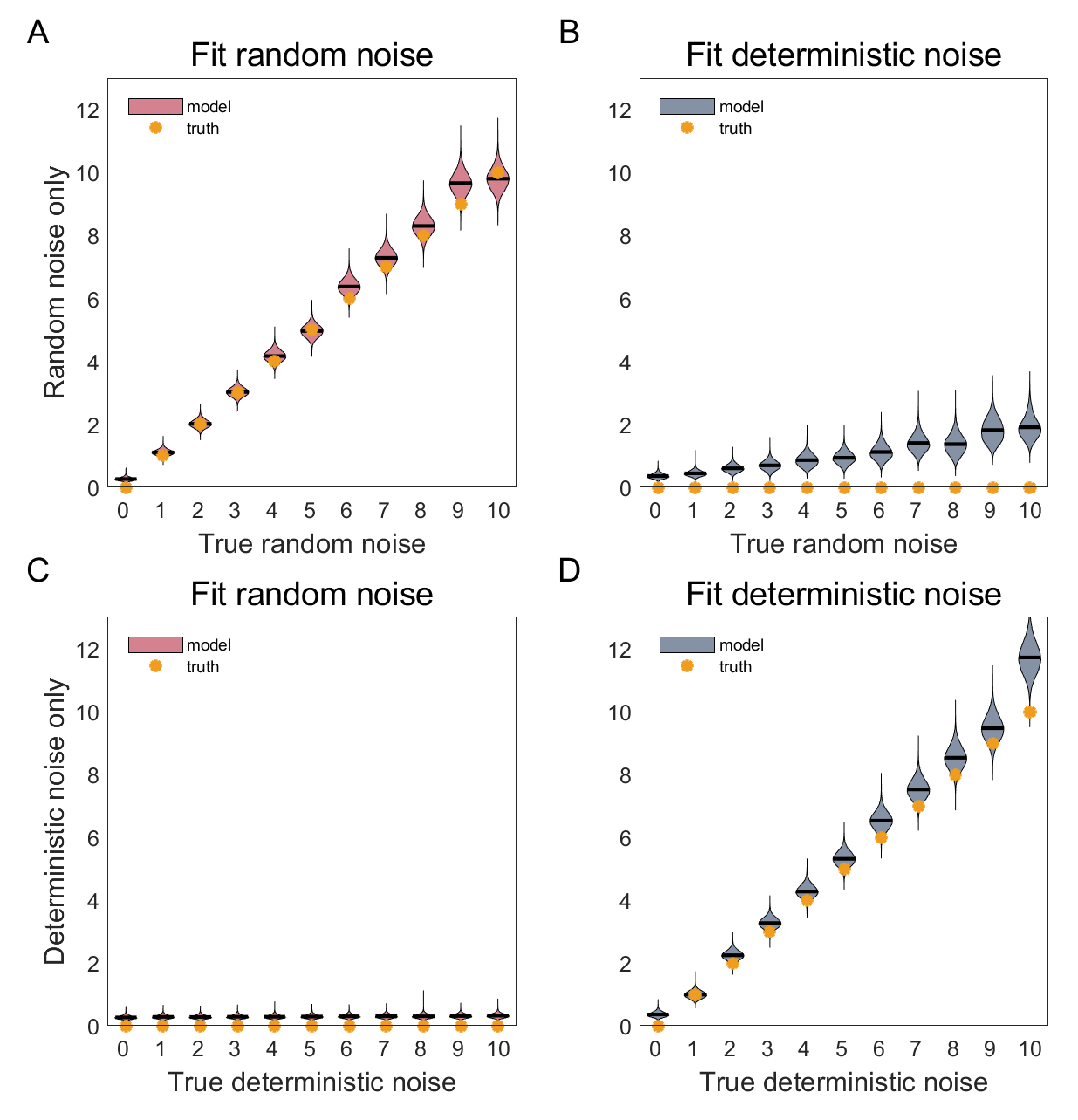

Supplement: S9 Fig — Parameter recovery over the posterior of random noise standard deviation, σran, and deterministic noise standard deviation, σdet, for purely random noise (top row) and purely deterministic noise (bottom row) games. (TIFF) [file pcbi.1014026.s009.tiff]

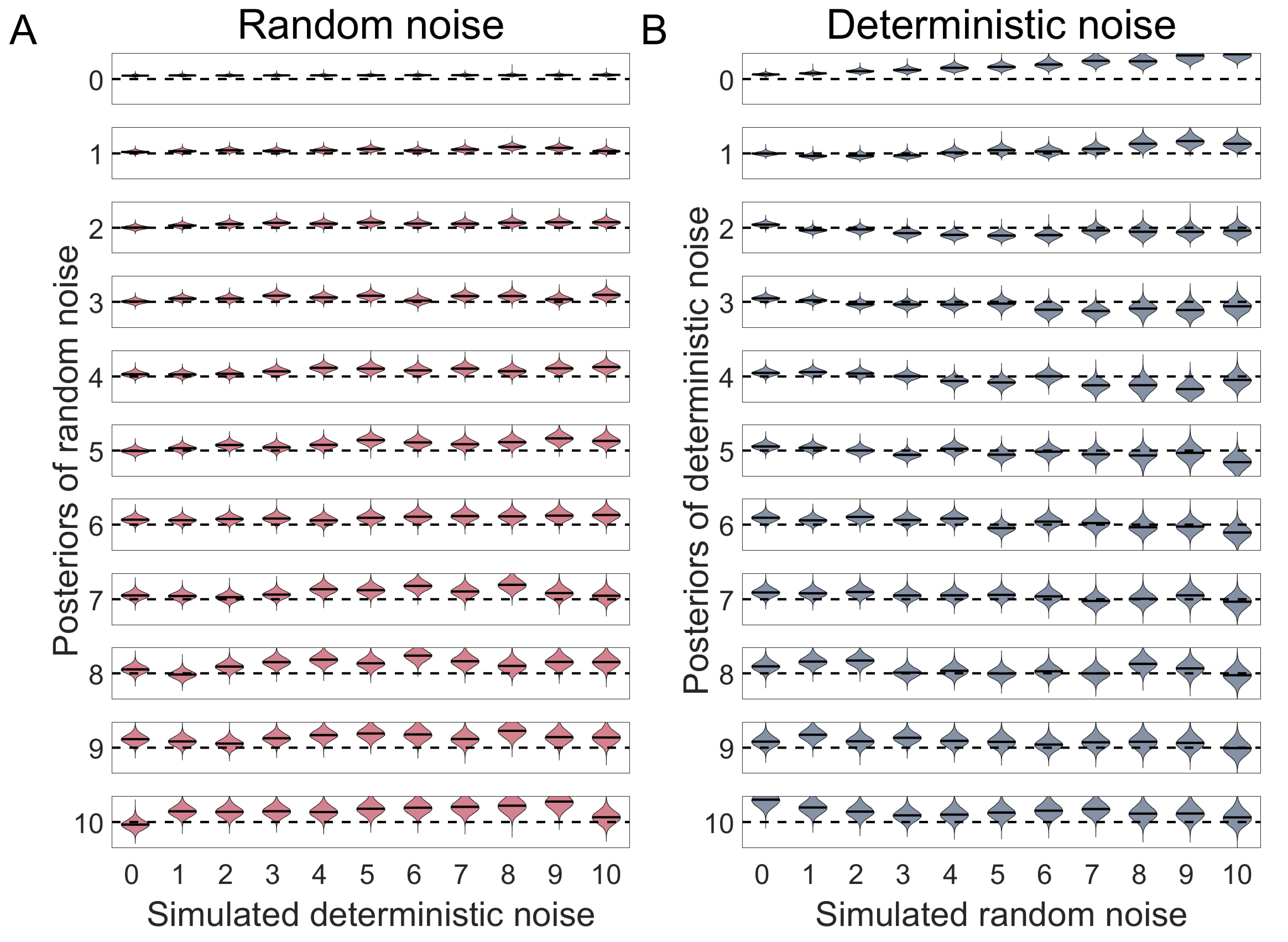

Supplement: S10 Fig — A. Recovered posterior distributions of random noise. B. Recovered posterior distributions of deterministic noise. For both A and B, from the top row to the bottom row, the true noise standard deviation that is used in the simulations go from 0 to 10. The y limit of each panel is 4 (+/- 2 from the true value). Our model did a relatively good job in recovering all combinations of deterministic and random noises. (TIFF) [file pcbi.1014026.s010.tiff]

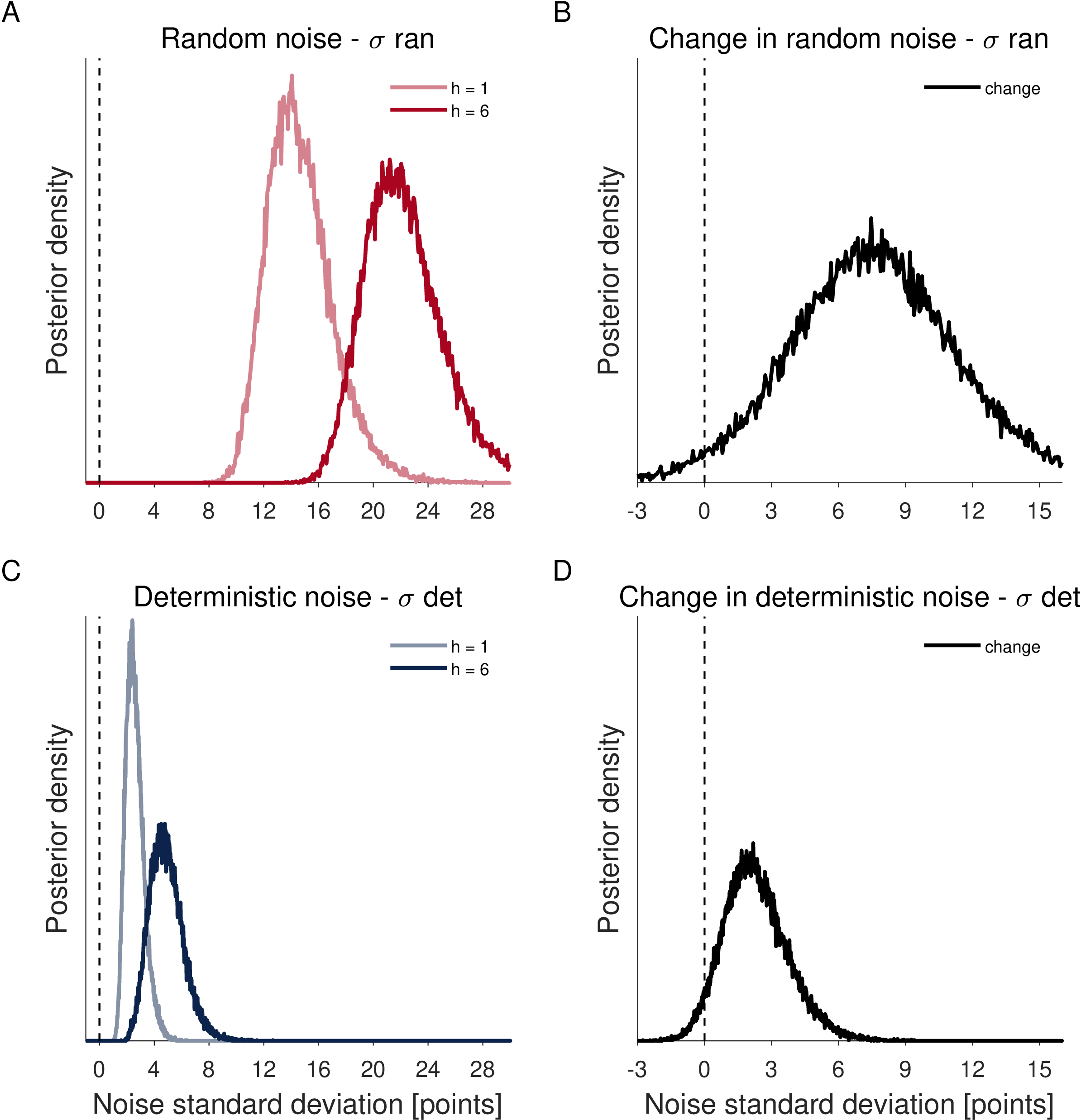

Supplement: S11 Fig — Both random (A, B) and deterministic (C,D) noises are nonzero (A, C) and change with horizon (B, D). However, random noise has both a greater magnitude overall (A, C) and a greater change with horizon (B, D) than deterministic noise. (TIFF) [file pcbi.1014026.s011.tiff]

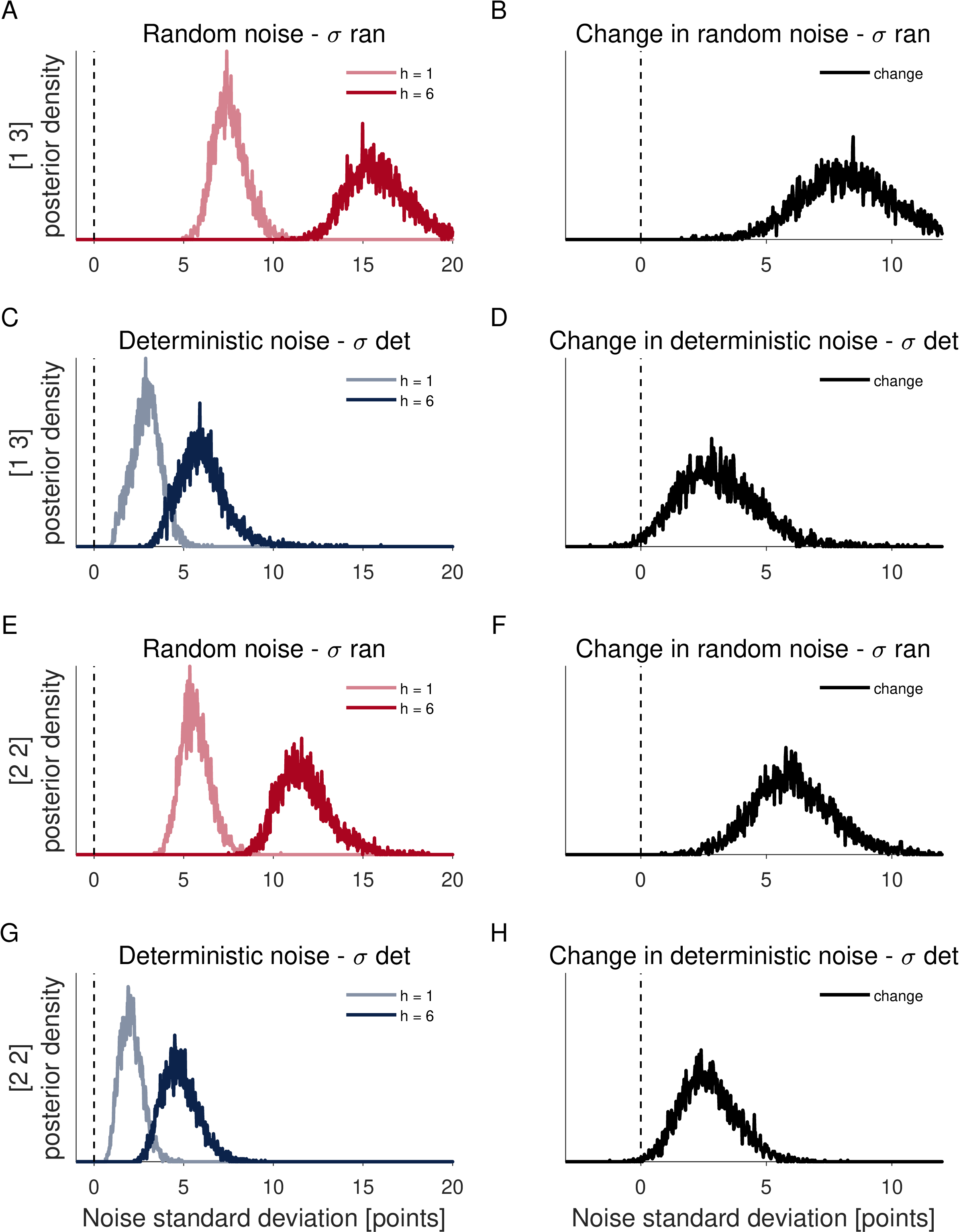

Supplement: S12 Fig — The posterior distributions over the group-level mean of the standard deviations of random and deterministic noise. Both random (A, E) and deterministic (C,G) noises are nonzero (A, C, E, G) and change with horizon (B, D, F, H). However, random noise has both a greater magnitude overall (A, E) and a greater change with horizon (B, F) than deterministic noise. Moreover, both random and deterministic noises have a greater magnitude in [1 3] compared to [2 2] conditions. (TIFF) [file pcbi.1014026.s012.tiff]

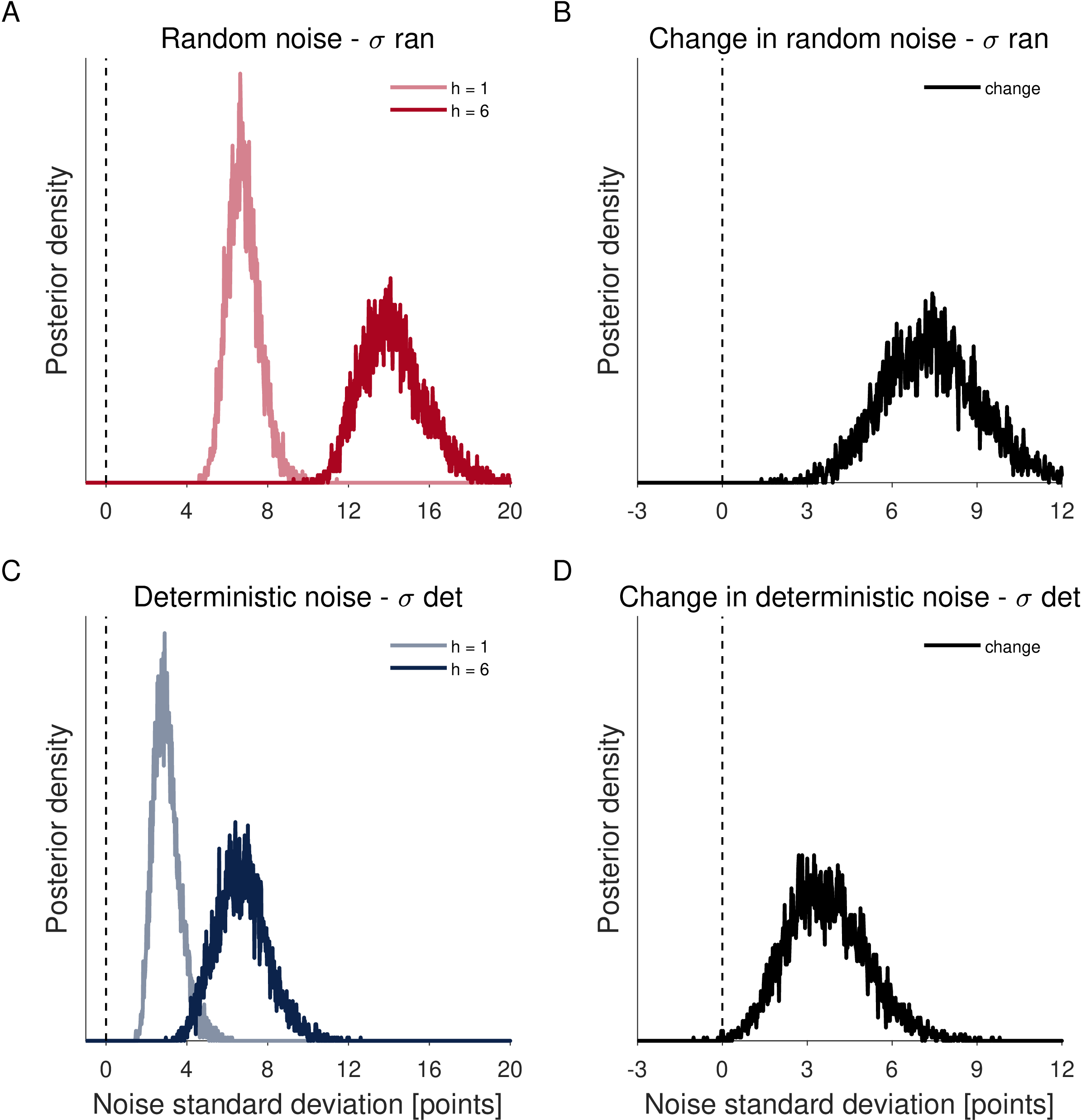

Supplement: S13 Fig — The posterior distributions over the group-level mean of the standard deviations of random and deterministic noise. Both random (A, B) and deterministic (C,D) noises are nonzero (A, C) and change with horizon (B, D). However, random noise has both a greater magnitude overall (A, C) and a greater change with horizon (B, D) than deterministic noise. (TIFF) [file pcbi.1014026.s013.tiff]

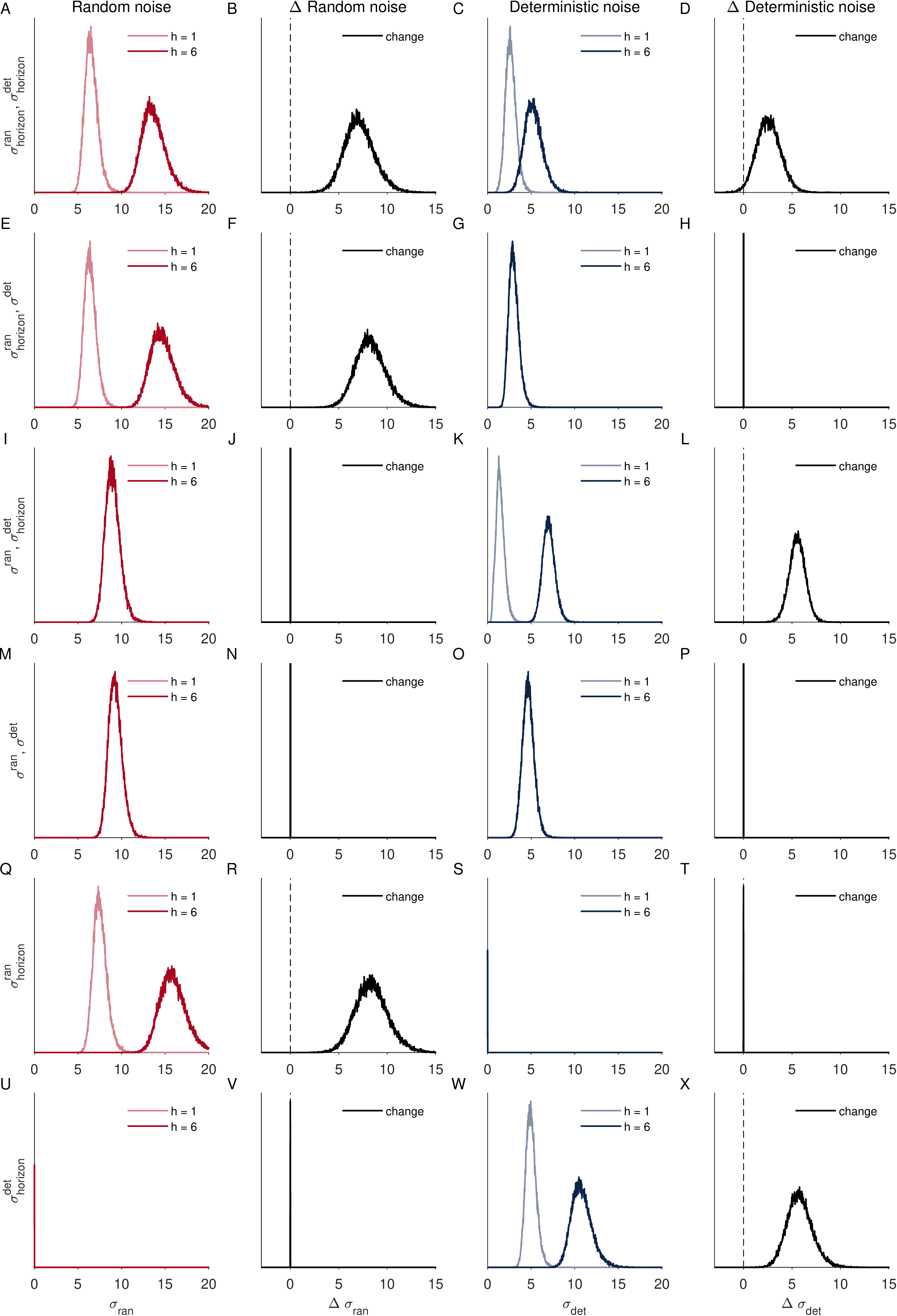

Supplement: S15 Fig — Each row is one model. These models varied in whether deterministic σdet and random noise σran are present or not and whether either types of noise is dependent on horizon (subscript denotes the dependence on horizon). (TIFF) [file pcbi.1014026.s015.tiff]

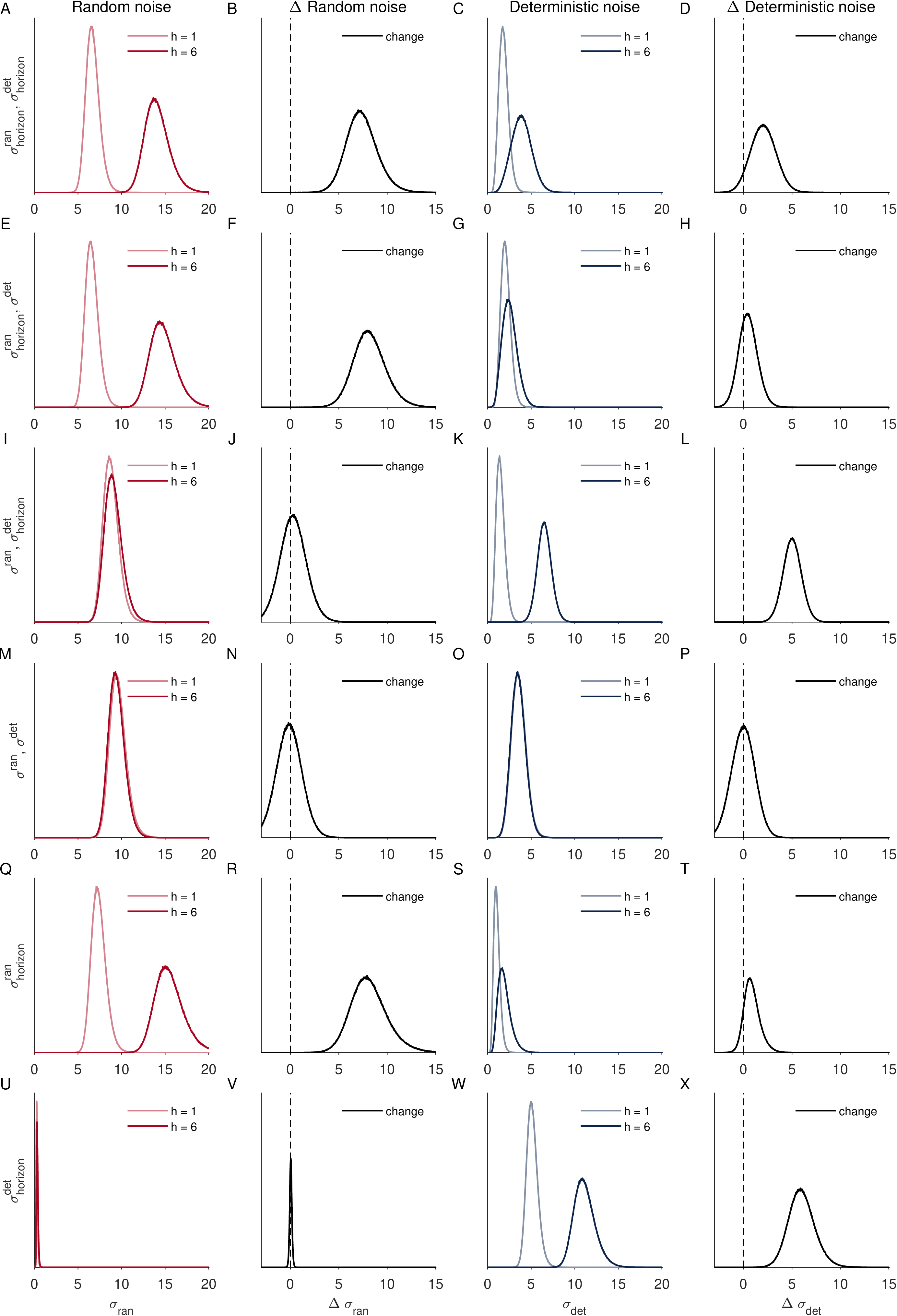

Supplement: S16 Fig — Our model qualitatively captures whether deterministic and random noise are present or not and whether either types of noise is dependent on horizon. A-D. both deterministic and random noise are horizon dependent, E-H. only random noise is horizon dependent, I-L. only deterministic noise is horizon dependent, M-P. neither random nor deterministic noise is horizon dependent, Q-T. only deterministic noise is assumed to be present, U-X. only random noise is assumed to be present. (TIFF) [file pcbi.1014026.s016.tiff]

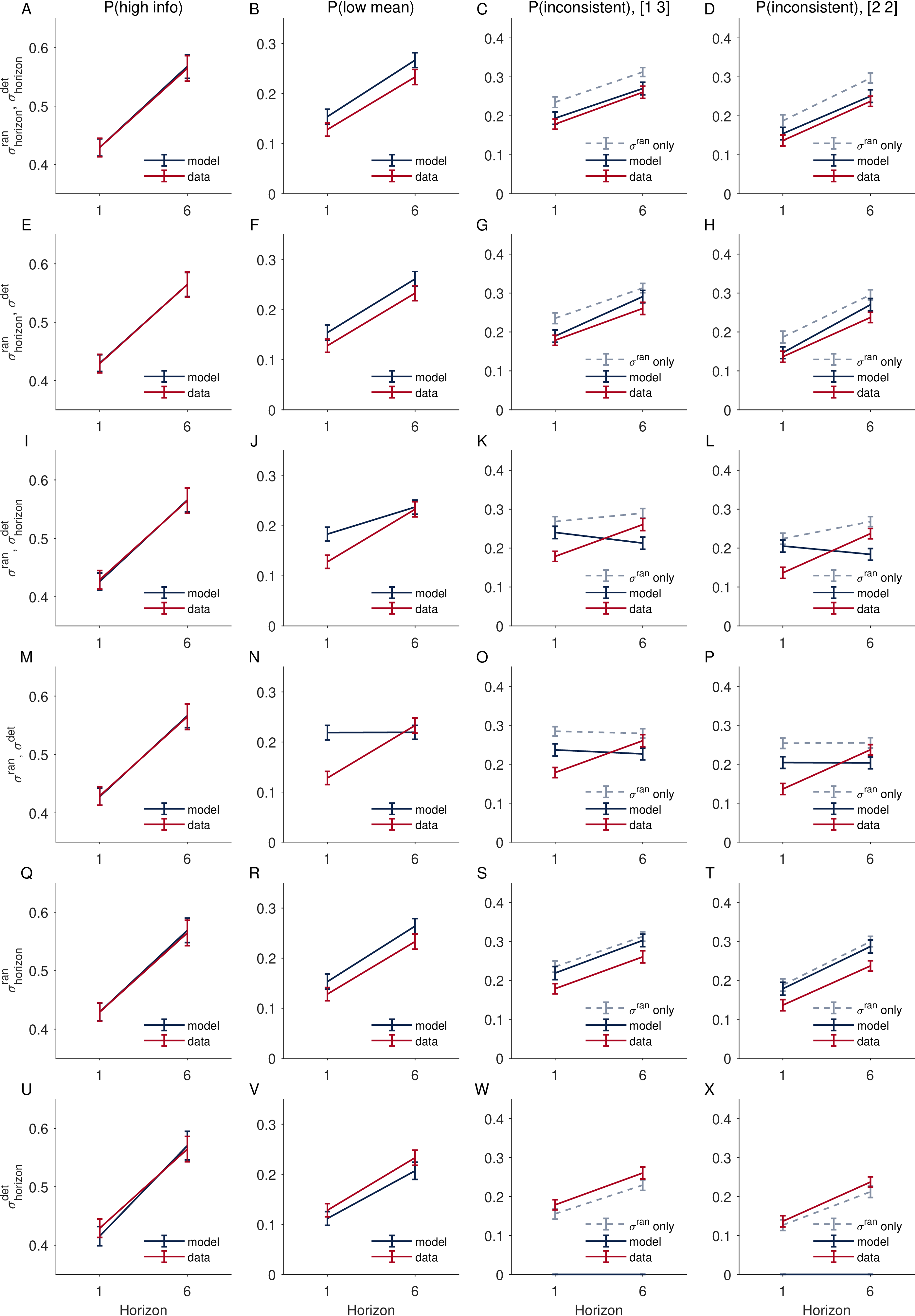

Supplement: S17 Fig — A-D. both deterministic and random noise are horizon dependent, E-H. only random noise is horizon dependent, I-L. only deterministic noise is horizon dependent, M-P. neither random nor deterministic noise is horizon dependent, Q-T. only deterministic noise is assumed to be present, U-X. only random noise is assumed to be present. (TIFF) [file pcbi.1014026.s017.tiff]

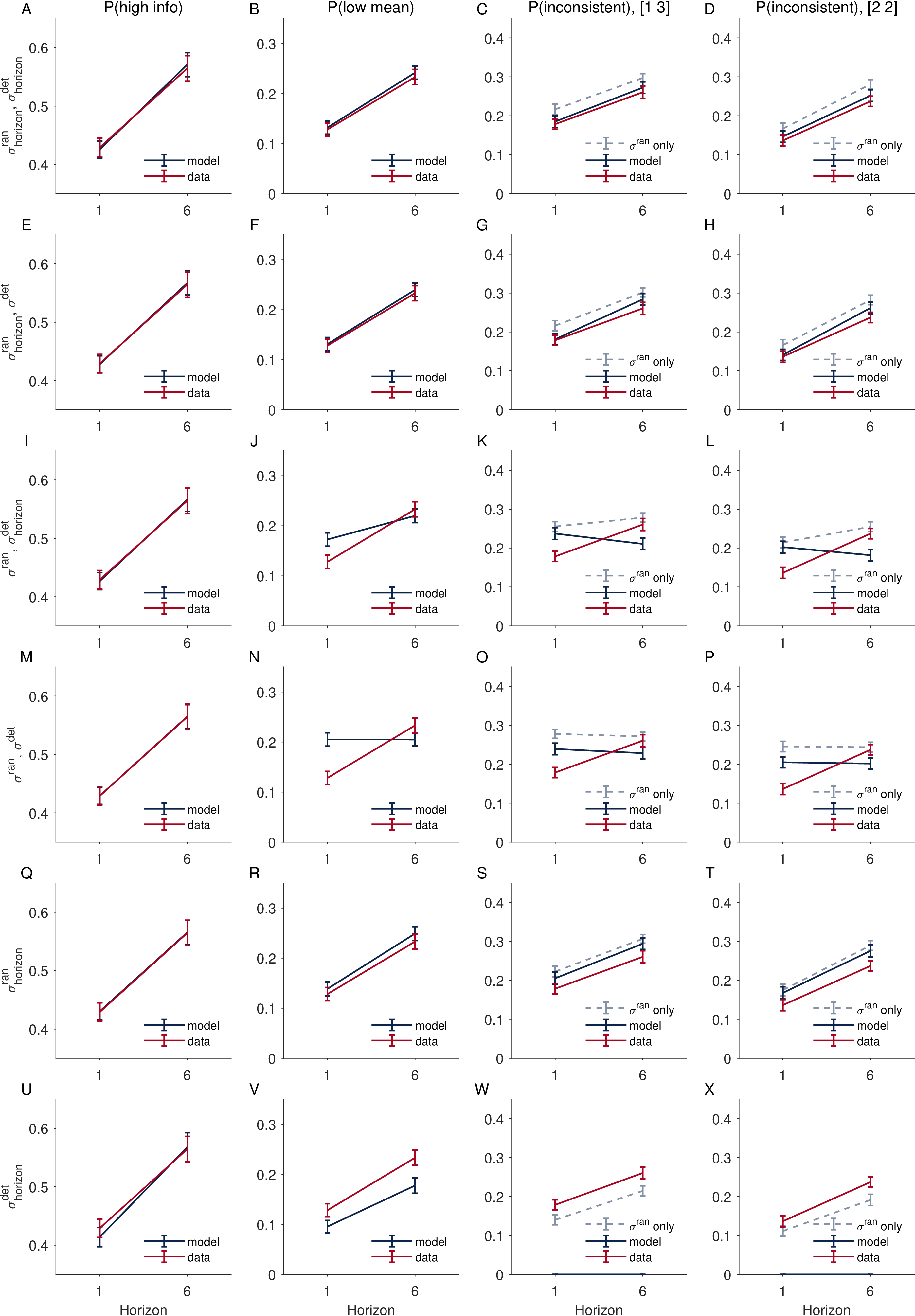

Supplement: S18 Fig — A-D. both deterministic and random noise are horizon dependent, E-H. only random noise is horizon dependent, I-L. only deterministic noise is horizon dependent, M-P. neither random nor deterministic noise is horizon dependent, Q-T. only deterministic noise is assumed to be present, U-X. only random noise is assumed to be present. (TIFF) [file pcbi.1014026.s018.tiff]
