# Supplementary material for: Separating random and deterministic sources of computational noise in explore-exploit decisions
Source: PLoS Comput Biol. 2026 Mar 17;22(3):e1014026. doi: 10.1371/journal.pcbi.1014026 (PMC13020999; doi:10.1371/journal.pcbi.1014026)
Supplement: S1 Text — (PDF) [file pcbi.1014026.s019.pdf]

Supplementary Materials: Separating random and deterministic sources of computational noises in explore-exploit decisions

Siyu Wang[1] and Robert C. Wilson[1,2,3,4,5]

[1]Department of Psychology, University of Arizona, Tucson, Arizona, United States of America

[2]Neuroscience and Physiological Sciences Graduate Interdisciplinary Program, University of Arizona, Tucson, Arizona, United States of America

[3]Cognitive Science Program, University of Arizona, Tucson, Arizona, United States of America

[4]School of Psychology, Georgia Institute of Technology, Atlanta, Georgia, United States of America

[5]Center of Excellence for Computational Cognition, Georgia Institute of Technology, Atlanta, Georgia, United States of America

Contents

1 Model validation analyses **3**

1.1 Deterministic noise captures known deterministic processes excluded from the model . . . 3

1.2 Frequentist coverage analysis 4

1.3 Parameter recovery of the subject-level parameter fits 5

1.4 Parameter recovery of arbitrary combinations of deterministic and random noises 6

2 Alternative models **7**

2.1 Reduced variations of the two-noise model 7

2.2 Evaluate our model's ability to detect horizon-dependent changes in random and deterministic noises 8

2.3 Posterior checks for alternative models 9

1 Model validation analyses

1.1 Deterministic noise captures known deterministic processes excluded from the model

We checked if our fitted deterministic noise could indeed capture unobserved deterministic process that was not accounted for by the decision model. We test this by leaving out one known deterministic process from the decision model, and ask if our method could recover that known deterministic process as deterministic noise. In particular, we fit a reduced version of our model that only considers reward and ignores the influence of uncertainty condition and spatial bias on explore-exploit decisions.

$$\Delta Q = \Delta R + n_{det} + n_{ran}$$

Here, $\Delta Q, \Delta R, n_{det}, n_{ran}$ represent the same variables as in the full model (Equation 2 in the manuscript). If deterministic noise in our model can indeed capture unobserved deterministic processes that's missed by the model, then we would expect to see a higher level of fitted deterministic noise in the reduced model compared to in the full model, whereas the level of random noise should remain unchanged. By comparing the fitted posterior distributions over the group-level means of the deterministic and random noise parameters σ_{det} and σ_{ran}, as expected, we observed an increase in deterministic noise and no change in random noise between the reduced and the full model (S4 Fig). This suggests that our model is capable of detecting missing deterministic processes.

1.2 Frequentist coverage analysis

We next evaluated our hierarchical Bayesian analysis procedure using the 'frequentist coverage analysis'. In the coverage analysis, we simulated choices with the fitted parameters from the Hierarchical Bayesian analysis, and then re-fit the model to the simulated choices to see whether we can recover the parameters (S5 Fig). The simulation and re-fitting was repeated for 200 times. Then we counted out of the 200 repetitions how many times the true parameter that we simulate the choices from lies in the fitted 95% confidence interval (S6 Fig). This ratio will be referred to as the coverage rate. If our model fitting is reliable, then the fitted confidence interval should cover the true parameter for more than 95% of the simulations. For random noise, the coverage rate is 100% for both horizon 1, horizon 6, and the horizon difference. For deterministic noise, the coverage rate is 66% for horizon 1 and 69% for horizon 6. By comparing the posterior distributions of parameters that were used to generate simulations and the posterior distribution of recovered parameters, it is clear that our model systematically underestimates deterministic noise (S5 Fig, S6 Fig). Despite the underestimation of deterministic noise in both horizons, we could still reliably detect the horizon changes of deterministic noise (coverage rate is 97%). This is because the underestimation of deterministic noise is partially canceled out when the difference is taken between horizons. For random noise, our model fitting procedure yields a faithful recovery. However, there is a conceptual limitation. Because random noise is modeled as non-stimulus-driven noise, it can include both true stochastic random noise and possible deterministic noises which do not depend on the stimuli. Because of this, our random noise estimate provides an upper bound of true 'random noise' induced by intrinsic stochastic processes in the brain.

1.3 Parameter recovery of the subject-level parameter fits

Next, we tested the ability of our model fitting procedure to recover parameters from simulated data at the subject level (S7 Fig, S8 Fig). The correlations between the true vs fitted parameters are significant across participants for all parameters ($p < 0.001$). The strength of correlation between simulated and fit values are strong for both deterministic noise and random noise. Despite the strong inter-subject correlations, we again observed a systematic underestimation of σ_{det} (S7 Fig, S8 Fig).

Overall, we are able to detect both deterministic and random noises using our model to a satisfactory extent. Our model provides a lower bound for deterministic noise and an upper bound for random noise. In addition, We see better parameter recovery for random noise than deterministic noise. This is likely because we effectively have half as many trials for deterministic noise. In particular, while we generate two samples of random noise for each repeated game pair, we only generate one sample of deterministic noise, which by definition is the same in both of the repeated games.

1.4 Parameter recovery of arbitrary combinations of deterministic and random noises

Lastly, in addition to testing how our model performs in parameter ranges around the actual fitted parameters, we tested the limitations of our models in arbitrary combinations of random vs deterministic noises. All combinations of random and deterministic noises with $0 \leq \sigma_{det} \leq 10$ and $0 \leq \sigma_{ran} \leq 10$ were tested. In a special case, we evaluated how our model performs when there is only random noise or only deterministic noise (S9 Fig). In the simulation with fully deterministic noise and 0 random noise, our model successfully recovered both random and deterministic noise (S9 Fig C, D), however in the simulation with fully random noise and 0 deterministic noise, although our model successfully recovered random noise, some small proportion of deterministic noise was falsely detected when they should instead be 0 (S9 Fig A, B). However, this phenomenon only exists when the true deterministic noise is 0, once the true deterministic noise is greater than 1, we don't observe this obvious inflation of deterministic noise anymore (S10 Fig). Apart from this, our model did a fairly good job in recovering all combinations of random and deterministic noises (S10 Fig).

2 Alternative models

2.1 Reduced variations of the two-noise model

In this section, we examined 6 model variants that varied in whether deterministic and random noise are present or not and whether either types of noise is dependent on horizon (Table A). Specifically, we tested the following 6 models (Note that the $\sigma^{ran}_{horizon}, \sigma^{det}_{horizon}$ model is our original full model).

Model	Deterministic noise	Random noise
$\sigma^{ran}_{horizon}, \sigma^{det}_{horizon}$	Horizon dependent	Horizon dependent
$\sigma^{ran}_{horizon}, \sigma^{det}$	Fixed	Horizon dependent
$\sigma^{ran}, \sigma^{det}_{horizon}$	Horizon dependent	Fixed
$\sigma^{ran}, \sigma^{det}$	Fixed	Fixed
$\sigma^{ran}_{horizon}$	Horizon dependent	None
$\sigma^{det}_{horizon}$	None	Horizon dependent

Table A. Variants of the model.

These models were fit to the data. The posterior distributions over the group-level means of the deterministic and random noise standard deviation σ_{det} and σ_{ran} (when they exist) in these model variants are shown in S15 Fig.

2.2 Evaluate our model's ability to detect horizon-dependent changes in random and deterministic noises

We then examined if our model can indeed qualitatively capture whether deterministic and random noise are present or not and whether either types of noise is dependent on horizon. To test this, we simulated choices from each of the 6 models, and then fit the simulated choices with our original full model. The simulation was repeated 50 times for each model. Indeed, we showed that our model can capture both the existence of random and deterministic noise, and whether each noise changes with horizon condition (S16 Fig), with only one exception that our model falsely detected a small fraction of deterministic noise when no deterministic noise was present (S16 Fig). This phenomenon was also examined and discussed in the section 1.4 above.

2.3 Posterior checks for alternative models

To check whether all aspects of the model were necessary to reproduce the qualitative pattern of findings, we again simulated choices using fitted parameters from these models and repeated the model-free analysis on the simulated data. As shown in S17 Fig, only one of these alternative models, where random noise is horizon dependent but deterministic noise is not, can capture most of the qualitative features, including the increase in p(high info), p(low mean) and p(consistent). However, the quantitative fit to the data is not as good (S17 Fig). Moreover, when comparing p(consistent) with the theoretically predicted p(consistent) for pure random noise (see analysis in Fig 3), only the fully model is statistically significant in both horizon conditions. Out of 50 simulations, the best alternative model only shows statistical significance in half of the simulations in Horizon 6, and fails on the other 50% of the times. Because deterministic noise is not allowed to change with horizon in this alternative model, in horizon 6, deterministic noise is not large enough compared to the magnitude of random noise, to make p(inconsistent) lower than the random-noise-only prediction.

In our model, the subject-level noises are assumed to follow a gamma distribution (to ensure positiveness), the posteriors are right skewed (maximal likelihood estimation or mode is smaller than the mean), Because of the skewness, simulating data with the “mean” is noisier than simulating with the true distribution. Simulating from the true distribution requires taking expected value over all possible noise values, for ease of implementation, we simulated data by taking random samples from the posterior distribution (instead of using the mean). The simulation was repeated 50 times and then averaged (S17 Fig). Simulation using the ”mode” or maximal likelihood estimation was also shown here (S18 Fig) and produces closer fit to the data.
