# Supplementary material for: Separating random and deterministic sources of computational noise in explore-exploit decisions
Source: PLoS Comput Biol. 2026 Mar 17;22(3):e1014026. doi: 10.1371/journal.pcbi.1014026 (PMC13020999; doi:10.1371/journal.pcbi.1014026)
Supplement: Attachment — Submitted filename: PlosCB_revision_v1.docx [file pcbi.1014026.s020.docx]

We would like to thank the reviewers for carefully reading our manuscript and providing detailed and useful comments. We have addressed all comments and believe that the additional details and analyses have strengthened the manuscript.

Reviewer's Responses to Questions

**Comments to the Authors:
Please note here if the review is uploaded as an attachment.**

Reviewer #1: This manuscript describes a cognitive modeling study of human choice behavior in a well-known task (the Horizon task) developed by the last author. In this study, the authors ask whether part of the decision noise that is captured by the standard cognitive model used to fit human choices is not genuinely random (unpredictable), but rather deterministic (predictable). To split decision noise in the Horizon task into random and deterministic terms, the authors apply a repeated-trial approach that has been described and applied in tightly connected contexts to show that at least 14% of random exploration is due to deterministic biases that affect choices in the same way across repetitions of the same trial. More interestingly, the authors report that not only random, but also deterministic sources of noise increase with horizon length, an effect that the authors discuss in terms of a decrease in the decision gain of the reward term (rather than an increase in the decision gain of the information bonus term).

I found the manuscript to be very well written and structured in terms of analyses and results, the authors adequately motivate their study in the introduction and the (repeated-trial) approach they have chosen to use to address their research question. The methods also appear well suited to provide statistical support for their findings, and their discussion of the results (in particular the joint increase of random and deterministic noise components with horizon length) is interesting from a cognitive perspective. I nevertheless have a few comments below which the authors should address in my opinion to make the manuscript stronger and better reflect the existing literature that has used in recent years the exact same approach - in very similar contexts - to decompose choice variability into random and deterministic components.

**Comment 1.1:** * The current version of the manuscript is missing recent references (copied below) that describe (ref. 1 and 2) and apply/discuss (ref. 3 and 4) the same (repeated-trial) approach to cognitive problems that are tightly connected to the one studied here. These references should be cited in the revised manuscript to provide additional theoretical (and empirical) background about this bias-variance separation approach. They would also provide additional findings that can be used to discuss the findings obtained by the authors in the present study:

1/ Wyart V, Koechlin E (2016) Choice variability and suboptimality in uncertain environments. Current Opinion in Behavioral Sciences 11, 109-115. doi:10.1016/j.cobeha.2016.07.003

2/ Wyart V (2018) Leveraging decision consistency to decompose suboptimality in terms of its ultimate predictability. Behavioral and Brain Sciences 41, e248. doi:10.1017/S0140525X18001504 - Commentary on Rahnev D, Denison RN (2018) Suboptimality in perceptual decision making. Behavioral and Brain Sciences 41, e223. doi:10.1017/S0140525X18000936

3/ Findling C, Skvortsova V, Dromnelle R, Palminteri S, Wyart V (2019) Computational noise in reward-guided learning drives behavioral variability in volatile environments. Nature Neuroscience 22(12), 2066-2077. doi:10.1038/s41593-019-0518-9

4/ Findling C, Wyart V (2021) Computation noise in human learning and decision-making: origin, impact, function. Current Opinion in Behavioral Sciences 38, 124-132. doi:10.1016/j.cobeha.2021.02.018

**Response 1.1:**

We thank the reviewer for bringing up these relevant references. Reference 3 was already cited in the original manuscript. We have added the other references to the introduction and the discussion.

**Comment 1.2:** * The authors have applied parameter recovery and posterior predictive checks to check whether their fitting procedure is capable of estimating parameter values, and of predicting the key features of the observed human choice behavior. These two procedures have been described as critically important by Wilson and Collins (2019, eLife) and by Palminteri, Wyart and Koechlin (2017, Trends in Cognitive Sciences - missing reference which should ideally be cited where posterior predictive checks are first described). They reveal that the deterministic noise term is underestimated by the fitting procedure, and that the best-fitting model overestimates p(low mean) and p(inconsistent) in the [2,2] condition - which is the most ‘basic’ condition without information bonus.

The authors do not discuss these biases of the fitting procedure, but it would be important to understand why it is the case. Could it be due to the hierarchical fitting approach used by the authors? Why did the authors choose this hierarchical fitting approach over and above a simpler, independent (subject-wise) fitting approach? The theoretical merits of a hierarchical fitting approach are clear and obvious, but could the authors employ the simpler subject-wise fitting approach to check that the same biases of the fitting procedure remain present (and therefore that they are not triggered by the hierarchical fitting approach)?

**Response 1.2:**

Non-hierarchical fits

The reason we chose the hierarchical approach was due to the difficulty of estimating deterministic noise which requires many trials across participants to properly converge. In our model, instead of using softmax to output a choice probability, to separately estimate deterministic and random noises, we had to sample both types of noises and had to sample a frozen noise for each repeated pair of games. This made it more challenging for the MCMC to converge with limited data, compared to fitting more commonly used softmax-based models.

In fact, if we use a non-hierarchical approach, the mean estimates of the subject-level noises remain at very large values due to the large variance in the posterior. We assumed a non-informative prior that spans a large range of possible noise values. Since the non-hierarchical fits rely only on the limited number of trials from each individual subject, and the amount of data per subject is not enough for the model to converge to meaningful values, the posteriors remain broad and we get unreasonably large noise estimates.

Bias in parameter recovery

Nevertheless, as requested by the reviewer, we performed parameter recovery on non-hierarchical fits to see if it resolves the bias in parameter recovery. In the above figure, the top row shows parameter recovery for hierarchical fits (note that noise estimate is between 0-40), and the bottom row is for non-hierarchical fits (note that noise estimate is up to 300), left column is for random noise, and right column is for deterministic noise. As you can see in the above figure, non-hierarchical fitting procedure does not fix the underestimation of deterministic noise. In fact, it also underestimates random noise.

We think the underestimation of deterministic noise is partially due to limited data. To properly estimate large noises, more data is needed. As we only have half as many trials for deterministic noise as for random noise, we don’t have enough data to reliably estimate large values of deterministic noise.

We acknowledged this limitation and stated in our manuscript that our method provides a lower bound for deterministic noise (as opposed to a faithful recovery).

Bias in posterior checks

We cited the reference suggested by the reviewer for posterior checks.

For the overestimation of p(low mean) and p(consistent) in [2 2] condition in posterior checks, we have identified two factors that led to the mismatch:

0. For comparison, we attach here the posterior check figure from the initial submission.

1. In our model, we assumed that the variances of deterministic and random noises are from a constant distribution for both the [1 3] and [2 2] information conditions. Since our model overestimates p(low mean), it suggests that we are overestimating noise in [2 2] condition. It is possible that people have higher noises in [1 3] condition compared to [2 2] condition, by assuming the [1 3] and [2 2] condition share the same noise distribution, it will lead us to overestimate noise in [2 2] condition.

To fix this, we fit a variant of our model in which we separately estimate the variances of random and deterministic noises in [1 3] and [2 2] conditions. Indeed, we observe higher overall noise level for [1 3] condition. When performing posterior checks with this model, the mismatch between data and model becomes smaller in the [2 2] condition.

2. In our model, the subject-level noises are assumed to follow a gamma distribution (to ensure positiveness), the posteriors are right skewed (maximal likelihood estimation or mode is smaller than the mean), in the original analysis, we simulated data from each participant using the mean of the subject-level posterior for both deterministic and random noises, however, because of the skewness, simulating data with the “mean” is nosier than simulating with the true distribution. Simulating from the true distribution requires taking expected value over all possible noise values, for ease of implementation, we simulated data by taking random samples from the posterior distribution (instead of using the mean). The simulation was repeated 50 times and then averaged, below is what we get. Indeed, the mismatch between data and model further decreased. (we adopted this method in the main text, as this is the closest to the data generation process in the model)

If we use the mode, which corresponds to the maximal likelihood estimation estimate, we can get the model simulation to closely match the data:

**Comment 1.3:** * The authors appear to take for granted that the joint increase of random and deterministic sources of noise with horizon length is genuine and not caused by a limitation of the fitting procedure. I tend to agree with their interpretation, but it would be very useful to provide additional empirical evidence in the main text that this joint increase is indeed genuine. The parameter recovery approach illustrated in Figure 4 should include panels of figures found in the Supplementary Materials which show that the fitting procedure is capable of correctly recovering arbitrary combinations of random and deterministic sources of noise. A simpler and more compact test, which the authors should perform and plot as a new panel of Figure 4, would be to plot the confusion matrix arising from the parameter recovery procedure (Figure 4 only shows what corresponds to the diagonal of the confusion matrix). It is indeed critically important that simulated ground-truth values of random noise do not correlate with best-fitting values of deterministic noise (and vice versa).

This would require to fit choice behavior using a non-hierarchical, subject-wise fitting approach, or to complexify the hierarchical fitting approach to estimate the covariance between random and deterministic sources of noise. Both control analyses would be valid, and I let the authors choose whichever approach they find most appropriate for their data. This would provide empirical evidence (already available to the authors since they have performed a parameter recovery analysis) that random and deterministic sources of noise can indeed be reliably separated, and therefore that the joint increase of the two forms of noise with horizon length is genuine. This type of control analysis have been performed in a recent study, in case this is helpful:

Lee JK, Rouault M, Wyart V (2023) Adaptive tuning of human learning and choice variability to unexpected uncertainty. Science Advances 9, add0501. doi:10.1126/sciadv.add0501

**Response 1.3:**

To show that the joint increase of random and deterministic sources of noise is not caused by a limitation of the fitting procedure, following the reviewer’s advice, we calculated the correlation between ground-truth values of random noise, and best-fitting values of deterministic noise (and vice versa). Ground-truth values are shuffled best-fit parameters.

As expected, ground-truth random values do not correlate with recovered deterministic noises, showing that the increase of deterministic noise with horizon is genuine and not a by-product of increase of random noise, and vice versa.

Alternatively, in the supplemental materials, we also performed an analysis in which we simulated data where we have ground truth about whether random or deterministic noises change with the horizon condition. We fit our model to the simulated data and our model can faithfully detect the existence of horizon changes in both random and deterministic noises only when they exist, for simulated data. (See Supplemental Fig. S16)

Reviewer #2: In the manuscript entitled 'Separating random and deterministic sources of computational noise in explore-exploit decisions', Siyu Wang and Robert C. Wilson present an extension of the Horizon task by Wilson and colleagues (2014) to investigate whether random exploration in human decision-making is driven by stochastic processes in the brain or by some unobserved deterministic process. The task is extended so as to disentangle deterministic noise from random noise by presenting a situation where, unbeknownst to them, participants are presented with the exact same choice twice. This enables the authors to estimate a lower bound on the amount of variability that is deterministically driven by the stimulus and an upper bound on the amount of variability that is random. They found evidence that at least 14% of the variability in random exploration in their task can be accounted for by deterministic processing of the stimulus.

**Comment 2.0:** The topic of this research is very interesting, timely and of importance to the community. The maths behind the computational models and the analyses seem correct and are elegantly developed. But at the end of the reading I am left only partially satisfied. The starting important question is: where does the 'random' noise identified by Wilson et al. 2014 come from? But the article arrives to the conclusion that there's around 14% of explainable random noise on a task where there are important limitations to the protocol (see detailed comments below), and without really finding explanations of where do those 14% of deterministic noise come from, not why participants' choices tend to be repeated. This would require to look at when random (or deterministic) choices occur. For example, when the average rewards displayed by the bandits are close (this is taken into account in the model), when the uncertainty displayed on one of the two bandit arms is higher than for the other (risk seeking or risk averse behavior), when there is an attractor point (a higher reward) for the choice that is sub-optimal. The authors themselves admit this in the discussion: 'As a result, from both a conceptual and methodological perspective, it is possible that the remaining 86% of the decision noise that is not stimulus-driven noise, could be deterministic. ' I wish they'd develop more hypotheses on this.

**Response 2.0:** We sincerely thank the reviewer for carefully reading our manuscript and giving us detailed and constructive feedback.

Before we address the reviewer’s comments point by point, we would like to make a general comment and clarify the scope and focus of our paper. While we understand the reviewer’s “partial satisfaction” that we did not attempt to explain the exact source of the 14% of deterministic noise and the 86% of random noise, we would like to point out that this is almost intentional and is considered an advantage of our method. Instead of making hypotheses about the exact task-specific sources of deterministic and random noises, we took an alternative approach in this paper and developed a novel Bayesian approach which allowed us to separate deterministic noise from random noise without explicitly specifying or knowing their sources.

Although we have some ideas about where the 14% of deterministic noise might come from (e.g., motor sequence patterns of key presses during the forced-play trials), we leave this pursuit of task-specific modeling the deterministic noise for future work, since it will not affect the main message of our paper.

Overall, I feel that some control analyses and ways to discard alternative interpretations are needed.

ANALYSES

**Comment 2.1:** I am intrigued by the negative information bonus A in the model-based analyses for Horizon=1 (Figure 2D). Is it significantly different from 0? If yes, does this mean that participants are even avoiding uncertainty (risk aversiveness) in that case? What would be the implications of this?

**Response 2.1:** Yes, the negative information bonus in horizon 1 is significantly different from 0. This phenomenon was previously reported and interpreted in the original Horizon Task paper (Wilson et al., 2014). One of the main contributions of the Wilson et al., 2014 paper, was to use the horizon manipulation to separate “risk preference” (negative information bonus in Horizon = 1 which reflects uncertainty aversion) from “directed exploration” (changes in information bonus between Horizon 1 and 6 which reflects information-driven exploration).

**Comment 2.2:** I do not fully understand how the plotted values for the 'pure random noise prediction' (i.e., 'random noise only' in the figures) and 'deterministic noise only' in Fig. 3, Suppl. Fig. S2 and S3 were computed. If these correspond to theoretical values for the choice inconsistency for the purely deterministic and purely random noise cases, as formalized pages 11 and 12, then I don't understand why these values have standard deviations and vary so much between figures: in the [2 2] condition of Figure 3, the plotted mean of random noise only are <0.2 for Horizon 1 and <0.3 for Horizon 6, while in the [2 2] condition of Suppl. Fig. S3, they are >0.2 for Horizon 1 and >0.3 for Horizon 6. I expect that the simulated data in the [2 2] condition of Suppl. Fig. S3 are significantly different from the 'pure random noise prediction' (i.e., 'random noise only') in the [2 2] condition of Figure 3. Isn't it a problem and shouldn't the authors solve it here?

**Response 2.2:** I am happy to clarify this:

1. How was “pure random noise prediction” computed?

“pure random noise” refers to the assumption that participants treat the repeated games independently (there is zero deterministic noise). Under this assumption, considering a single game, if we know the probability that a participant chooses option A, then the probability of making consistent/same choices in repeated games would be:

$$P\left( consistent \right)= p\left( choose A twice or choose B twice \right)=p\left( choose A \right)^{2}+ p\left( choose B \right)^{2}$$

$$= p\left( choose A \right)^{2}+(1-p{\left( choose A \right))}^{2}$$

If we define option A to be the option that has a lower mean reward from the first four forced-choice trials, then the above formula becomes:

$$P\left( consistent \right)=p\left( low mean \right)^{2}+(1- p{\left( low mean \right))}^{2}$$

The “pure random noise prediction” refers to the theoretical prediction of p(consistency) using the above formula. When considering all games, we predict p(consistent) based on the empirical percentage of choosing the low mean option, p(low mean), based on each participant’s behavior.

2. Why do these theoretical values have error bars and vary between figures?

Since we use p(low mean) from each participant’s behavior, we calculate the theoretical p(consistent) for each participant, and the error bar is the standard deviation of the predicted p(consistent) across participants.

The reason Figure 3 and Figure S3 have very different y values is because Figure 3 is from data (where both deterministic and random noises presumably exist), and Figure S3 is from our simulation from a model that has only random noise and zero deterministic noise.

3. Explanation of Supplementary Fig. S3:

The point of Figure S3 is to empirically validate our theoretical calculation. This is not a posterior check analysis, we did not simulate from the best-fit parameters. Instead, we simulated data from a model that assumes 0 deterministic noise and all random noise. Our claim that deterministic noise exists comes from the fact that the data line significantly deviates from the theoretical pure random noise line in Figure 3. As a control, if we simulate data from a model that only has random noise, we expect the data line to be not statistically different from the theoretical predicted line, and that is exactly what we see in Fig S3 (pasted below).

**Comment 2.3:** I think the model validation analyses in supplementary data are very useful and well-performed. Nevertheless, shouldn't the spatial bias term be kept in all model versions to make them comparable? Could the authors quantitatively show how much the spatial bias term contributes to explaining participants' behavior? In Suppl. Fig. S7, it seems difficult to recover the spatial bias parameter with the parameter recovery method. Why is that so?

**Response 2.3:** Spatial bias was in fact kept in all model versions. We included spatial bias as it was included in the standard Horizon Task behavioral model proposed in Wilson et al., 2014.

In practice, participants do not have spatial biases significantly different from 0. Since spatial bias term was small (around 0), it was relatively more difficult to recover. In parameter recovery analysis, we simulated data with the best-fit parameters from the data, so the simulated bias terms were small (close to 0) to begin with, making the recovery difficult.

**Comment 2.4:** Moreover, it would be useful to give the reader a quick grasp of the summarized results by showing a model recovery matrix (as in Wilson & Collins 2019) with all nested versions of the full model (those in Table S1). Does the full model win when the simulations are generated by the full model? Does a reduced model without random noise win when the simulations are generated by the very same model? Conversely, does a reduced model without deterministic noise win when the simulations are generated by the very same model?

**Response 2.4:** Model recovery analysis as in Wilson & Collins (2019) would require an estimate of the data likelihood for each model. While this is doable for most cognitive models, we want to point out that this is not feasible for our model.

In a traditional cognitive model, usually a choice probability $p$ is computed based on input (e.g., reward history), and choice is sampled based on this probability. However, our model is not probabilistic, and is binary instead:

$$\Delta Q=\Delta R+A\cdot\Delta I+b+n_{det}+n_{ran}$$

Instead of using a softmax to model randomness in behavior (which only has random noise), in order to separate random and deterministic noises, we had to sample $n_{det}$ and $n_{ran}$ trial-by-trial using a MCMC procedure, and for each trial, choice is 1 if $\Delta Q>0$, and choice is 0 if $\Delta Q<0$. As a result, there is no easy way to get a data likelihood estimate and a model recovery analysis in the traditional way is not possible.

Instead, to show that our model is capable of correctly measuring both random and deterministic noises and their horizon-dependent changes, we performed the supplemental analysis shown in Supplementary Fig. S16 (also attached below). In this analysis, we simulated data from the 6 variants of the reduced models, and fit the full model to data generated from each of the reduced models.

The idea is that the full model should only detect the existence and horizon-dependent changes of random/deterministic noises when the data-generating model have them. For example, when we simulate from a model that deterministic noise is fixed and random noise increases with Horizon, and fit our full model to the simulated data, we expect to see that the recovered deterministic noise does not change with horizon, but random noise does. And that’s exactly what we see (panels E-H).

Figure S16. Our model qualitatively captures whether deterministic and random noise are present or not and whether either types of noise is dependent on horizon. A-D. both deterministic and random noise are horizon dependent, E-H. only random noise is horizon dependent, I-L. only deterministic noise is horizon dependent, M-P. neither random nor deterministic noise is horizon dependent, Q-T. only deterministic noise is assumed to be present, U-X. only random noise is assumed to be present.

**Comment 2.5:** Do I understand correctly that in the full model as well as in the reduced one, the random noise (sigma ran) and the deterministic noise (sigma det) come into play only during the first choices that are repeated during two games, and not during other first choices nor during 2nd, 3rd, etc. choices? Or instead are these two noise terms contributing to all decisions? Could the authors make this clearer in the manuscript, please?

**Response 2.5:** Yes. Since the first free choice (the first 4 choices are forced, this would be the 5^th^ choice in both H = 1 and H = 6 games) is the only choice that is comparable between Horizon = 1 and Horizon = 6 conditions, all of our analyses were performed on the first free choice. While these two noise terms should contribute to all decisions, our paper only modeled the first free choice.

In the results section, we had this sentence when describing the task

“*By contrasting behavior between short and long horizon conditions on the very first free-choice trial, when all else is equal, the Horizon Task allows us to quantify how behavior changes, when it is more valuable to explore*.”

When describing the methods, we modified the following paragraph, hopefully it is clearer now:

*“…we focus on just the first free-choice trial in each game, where the only thing that differs between the horizon conditions is the number of choices that participants will make in the future.* *Subsequent choices in Horizon 6 games were not analyzed.”*

**Comment 2.6:** Could the authors add a few sentences in the supplementary information to clarify that if the random noise increases in the reduced model (the one without information bonus), it leads to less choice consistency between repeated games, and that conversely if the deterministic noise increases, it leads to more choice consistency?

Does the deterministic noise in the reduced model capture and replace the effect that the information bonus produces in the full model?

**Response 2.6:** In general, increasing random noise leads to higher p(low mean) and lower p(consistent) between repeated games, and increasing deterministic noise leads to higher p(low mean) and higher p(consistent).

When removing information bonus from the full model, we created a case where we know the reduced model is missing an important source of deterministic noise (explained by information bonus). As a result, we expect to see an increase in deterministic noise and that’s what we saw (Supplemental Fig. S4, also attached above). The increased deterministic noises show that in the reduced model, there are higher unexplained variances (in the reduced model) that are predictable from the stimulus, compared to the full model. And the increased deterministic noise reflects the missing of the “information bonus” term.

**Comment 2.7:** The ‘posterior predictive check’ analysis is very nice and still rarely performed in the literature. Nevertheless, why are the model and data so different for p(low mean) (Figure 7B)? Is the difference significant? How can this be explained and interpreted?

**Response 2.7:**  We thank the reviewer for asking us to examine deeper the quantitative mismatch between model and data in posterior checks. For the overestimation of p(low mean) and p(consistent) in [2 2] condition in posterior checks, we have identified two factors that led to the mismatch:

0. For comparison, we attach here the posterior check figure from the initial submission.

1. In our model, we assumed that the variances of deterministic and random noises are from a constant distribution for both the [1 3] and [2 2] information conditions. Since our model overestimates p(low mean), it suggests that we are overestimating noise in [2 2] condition. It is possible that people have higher noises in [1 3] condition compared to [2 2] condition, by assuming the [1 3] and [2 2] condition share the same noise distribution, it will lead us to overestimate noise in [2 2] condition.

To fix this, we fit a variant of our model in which we separately estimate the variances of random and deterministic noises in [1 3] and [2 2] conditions. Indeed, we observe higher overall noise level for [1 3] condition. When performing posterior checks with this model, the mismatch between data and model becomes smaller in the [2 2] condition.

2. In our model, the subject-level noises are assumed to follow a gamma distribution (to ensure positiveness), the posteriors are right skewed (maximal likelihood estimation or mode is smaller than the mean), in the original analysis, we simulated data from each participant using the mean of the subject-level posterior for both deterministic and random noises, however, because of the skewness, simulating data with the “mean” is nosier than simulating with the true distribution. Simulating from the true distribution requires taking expected value over all possible noise values, for ease of implementation, we simulated data by taking random samples from the posterior distribution (instead of using the mean). The simulation was repeated 50 times and then averaged, below is what we get. Indeed, the mismatch between data and model further decreased. (we adopted this method in the main text, as this is the closest to the data generation process in the model)

If we use the mode, which corresponds to the maximal likelihood estimation estimate, we can get the model simulation to closely match the data:

**Comment 2.8:** I'll go into more details about risk averse and risk seeking behavior. In the model, discretizing information amounts is an approximation that may have important consequences in terms of explanatory power and interpretation of the results. In particular, setting a value of 0 for information in a situation [2 2] is bit unsatisfying: even if the four elements have been drawn from distributions with the same variance, we may have two values close together on the left and two far apart on the right. For example, if you have two identical values on one side and two different values on the other, it's very clear that there's information to be found on one side but not on the other. This is not taken into account in the model and falls under the heading of 'random' noise, which sounds somehow a bit absurd. Changing this in the model wouldn't require much effort. For example, replacing -1, 0, 1 by the variance differences. The variance of a single element is 0, so in [1 3] and [3 1] we go from -1 or 1 to the value of the variance of the 3 elements, with a plus or minus sign in front. And in the case [2 2] we go from 0 to a difference in variance that is probably small but potentially non-zero. This wouldn't change the model much, but it would directly change the interpretation (what if the 14% came from there?). Otherwise, it gives me the impression that the authors are a bit over-interpreting their results of a model that may not be ideal, and that the value of 14% doesn't mean much.

**Response 2.8:** We understand the reviewer’s comment.

1. We acknowledge that our model is simplified in the sense that we discretize information amounts and fail to account for information difference in [2 2] condition. We chose to present this simplified model in the main paper, as this model was the standard model used for the Horizon task, and was the model proposed in the original Horizon task paper (Wilson et al., 2014).
2. We want to point out that the information difference in [2 2] condition which we fail to capture in our model, falls in the category of “deterministic noise” as opposed to “random noise”, as the amount of information difference in [2 2] condition for each game would be identical between repeated games.
3. Per reviewer’s request, we have implemented the requested version of the model, in which we explicitly consider uncertainty in both [1 3] and [2 2] options by defining dI to be the variance differences between bandits. Our main findings did not change when using this model. Specifically, we see that both deterministic and random noises increase with horizon.

We also see that random and deterministic noises increase with horizon at similar rates using this alternative model:

In this model (model VAR), actually 18.1% of variances is explained by deterministic noise. Note that this is higher than the 14% for the original model with dI = -1, 0 or 1. This shows that the 14% of deterministic noise can not be explained simply by the variance differences in [2 2] condition. The fact that deterministic noise is higher in model VAR might suggest that model VAR is a worse fit to behavior compared to the original model. While the difference in variances theoretically accounts better for information differences in [2 2] conditions, it is possible that it numerically does not fit the data as well.

If we assume a uniform prior over all possible reward values and use the example trials to compute the variance of the posterior for each bandit. The number of plays (1, 2, or 3) has a much bigger influence than the specific reward outcomes. The variances of the posteriors are actually tightly clustered around 3 numbers (for 1 sample, 2 samples and 3 samples, respectively), regardless of which 1, 2 or 3 rewards were shown. This suggests that assuming dI = -1, 0 and 1 did not miss much in capturing the information difference between options in this particular task.

**Comment 2.9:** There is a contradiction between the sentence 'these reduced models fail to capture all qualitative patterns (Supplementary Figure S13)' in the main article, and the sentence 'As shown in Figure S13, only one of these alternative models, where random noise is horizon dependent but deterministic noise is not, can capture the full qualitative pattern of behavior.' in Supplementary Information. The main original should clarify that one of the alternative models does capture qualitative patterns. Moreover, the authors should clarify which quantitative measure they used to decide 'not as good' in sentence 'However, the quantitative fit to the data is not as good (Figure S13)' in Supplementary Information. To me it seems in Suppl. Fig. S13 that the difference between the two models (Fig. S13 A-D vs. Fig. S13 E-H) and the data is not significant. Again, I think that a model recovery analysis would be needed here.

**Response 2.9:** We apologize for the ambiguity in our language. To clarify, all qualitative patterns include:

1. p(high info) and p(low mean) increase with horizon (Wilson et al., 2014 findings)
2. p(consistent) increase with Horizon
3. p(consistent) is statistically different from the theoretical predicted value of p(low mean)

We want to clarify that only the full model captures all of these patterns. The best alternative model (Fig. S17 E-H) is quite close, as it captures patterns 1 and 2, but does not fully capture pattern 3 in Horizon 6.

For the full model, p(consistent) differs from the theoretical random noise prediction (gray dotted line) statistically, whereas in the best alternative model (row 2 here), p(consistent) is only different from the random noise prediction in about half of the simulations in Horizon 6 (out of 50 simulations), due to the inability to increase deterministic noise with Horizon in this model.

**Comment 2.10:** In the Discussion section, the demonstrations of how a change in reward processing could affect random and deterministic noise should be acknowledged as similar to the demonstration of how a change in reward processing could affect random noise in Cinotti et al. 2019 Scientific Reports, where it is written that when 'all Q-values are downscaled in the same proportion as the reward [and] When these values are plugged into the softmax process, the result is exactly equivalent to a decrease of the inverse temperature, again in the same proportion.'

**Response 2.10:** We have added the reference to the discussion section.

**Comment 2.11:** What if the participants had sometimes a good memory of having already been confronted with the same game + the bandit they had previously chosen, and deterministically decide to pick the other bandit so at to see which payout they obtain in this case? This would be deterministic exploration policy at the game level rather than at the bandit level, in contrast to the authors' interpretation as non-stimulus-driven random noise in explore-exploit decisions. The authors argue that two identical games in their task are 'separated by several minutes in time so as to avoid detection'. But how could they unsure that the repetition has never been detected? What would be the interpretation of their results if let's say at least a proportion of game repetitions had been detected by the participants? It seems to me that a way to address this problem would be to redo the task and ask 2 questions after each game's first free choice: have you already encountered the same game before? If yes, which bandit had you chosen the previous time? On the one hand, this would prompt people to know that there are game repetitions, which would increase their vigilance towards this feature and would increase their detection probability, on the other hand, this would enable to separate repeated games for which participants' accurately remembered their previous choice from those where they failed to remember. Another solution, so as not to bias participants' responses during the task would be to ask them to fill a questionnaire after, where they are asked whether they think they have encountered twice the same situation. This would be less ideal than the proposed variant of the task, but at least give an idea whether this was a problem or not in the present task. Have the authors asked the participants such questions during a post-task questionnaire?

**Response 2.11:** Unfortunately, we did not formally ask whether participants detected a repeat. However, we did interview participants after the experiment and asked them about what strategies they used during the task, and what they thought the experiment was about, and none of the participants mentioned noticing a repeat.

The reviewer is correct that our model does not consider game-level deterministic strategy.

1. Game-level strategies like memorizing repeated games will show up as random noise in our current model, just like other deterministic processes that are not a deterministic function of the current game stimulus.

Because of this, we made clear that our method provides a lower bound of deterministic noise.

2. We checked p(low mean) separated by the 1^st^ repeat versus the 2^nd^ repeat (panel A below), the idea is that if people can detect repeats and have a tendency to explore the alternative in the second repeat, they should on average pick the low mean option more in the 2^nd^ repeat. But that’s only true in Horizon 6, in horizon 1 they show the opposite.

As a control, we also did the same analysis but separated trials by the 1^st^ half of the experiment versus the 2^nd^ half of the experiment, regardless of whether it’s the 1^st^ repeat or 2^nd^ repeat (panel B). It looks very similar to Panel A. It is likely that the difference we see are due to early vs late phase of the experiment, and likely not due to repeats.

3. Conceptually, our model could naturally be extended to account for game-level deterministic strategy. Instead of treating “reward history within a game” as stimulus, the whole history of rewards across games can be treated as the stimulus. But this is beyond the scope of the current paper.

We have added this limitation to the discussion section.

LITERATURE

**Comment 2.12:** In the abstract, I suggest to replace 'recent work suggests that variability can actually be adaptive' by 'a long body of machine learning work suggests that variability can actually be adaptive'.

In the introduction, to be fair with the existing computational literature on adaptive decision noise, after the sentence 'It has recently been shown that humans appear to use random exploration and can increase decision noise when it is more beneficial to explore (Findling et al., 2019, Gershman, 2018, Wilson et al., 2014)', I suggest the authors add the following: , as has also been suggested in computational models of animal behavior (Doya 2002 Neural Networks; Khamassi et al., 2013 Progress in Brain Research).

**Response 2.12:** We have updated the introduction and abstract accordingly.

**Comment 2.13:** Typos
Page 8, a logistic distributions.
Page 12, in the both the.
Page 19, a fixed random motion stimuli -> stimulus.
Page 25, in the reference by Beck et al. 2012, there is a duplication of bibliographical information. Same thing for Findling et al., Tomov et al., Musall et al., Hogeveen et al., Ebitz et al., and Costa et al.
Page 8 of Suppl. Info. (Section 2.3) to recovery parameters -> to recover.

**Response 2.13:** We have corrected these typos, thanks for pointing these out.
